# Intranasal bovine β-defensin-5 enhances antituberculosis immunity in a mouse model by a novel protein-based respiratory mucosal vaccine

**DOI:** 10.1080/21505594.2022.2080342

**Published:** 2022-05-30

**Authors:** Zhengmin Liang, Hao Li, Mengjin Qu, Yiduo Liu, Yuanzhi Wang, Haoran Wang, Yuhui Dong, Yulan Chen, Xin Ge, Xiangmei Zhou

**Affiliations:** College of Veterinary Medicine, China Agricultural University, Beijing, China

**Keywords:** Tuberculosis, bovine β-defensin-5, mucosal immunization, T_RM_, antibody response

## Abstract

Respiratory mucosal immunization is an effective immunization strategy against tuberculosis (TB), and effective mucosal vaccines require adjuvants that can promote protective immunity without deleterious inflammation. Mucosal BCG (Bacille Calmette-Guerin) is effective, but it causes a severe inflammatory response in the lung. A novel less cytotoxic mucosal vaccine AH-PB containing *Mycobacterium tuberculosis* (Mtb) cell surface antigens Ag85A and HspX (AH), as well as polyinosinic-polycytidylic acid (Poly IC) and bovine neutrophil β-defensin-5 (B5) adjuvants were prepared, with the overarching goal of protecting against TB. Then, the immunogenicity and protective efficacy of these vaccines via the intranasal route were evaluated in a mouse model. Results showed that intranasal AH-PB promoted tissue-resident memory T cells (T_RMs_) development in the lung, induced antigen-specific antibody response in airway, provided protection against *Mycobacterium bovis* (*M. bovis*), conferred better protection than parenteral BCG in the later stage of infection, and boosted the protective immunity generated by BCG in mice. Moreover, both B5 and Poly IC were indispensable for the protection generated by AH-PB. Furthermore, intranasal immunization with AH-B5 fusion vaccines also provided similar protection against *M. bovis* compared to AH-PB. Collectively, B5-based TB vaccine via the intranasal route is a promising immunization strategy against bovine TB, and this kind of immunization strategy may be applied to human TB vaccine development. These findings highlight the potential importance of B5 as a mucosal adjuvant used in TB vaccines or other respiratory disease vaccines.

## Introduction

Tuberculosis (TB) is one of the most common causes of death annually. In 2020, approximately 9.87 million people were diagnosed with TB worldwide, of which 1.28 million people succumbed [1]. Notably, *Mycobacterium tuberculosis* (Mtb) and *Mycobacterium bovis* (*M. bovis*) are the two important pathogens that cause TB. *M. bovis*, the cause of bovine tuberculosis (bTB), exhibits a 99.9% sequence similarity to Mtb and has the ability to infect humans [2,3]. There was 1.3%–30.2% of human TB caused by *M. bovis* [4–6]. It will be difficult to achieve the goal of “The End TB Strategy” if bTB is not well controlled [3]. Bacille Calmette-Guerin (BCG), the only TB vaccine currently in use, provides protective immunity against disseminated TB in infants but has variable efficacy in adolescents and adults [7]. BCG also exhibits variable efficacy against bTB [8]. Therefore, this calls for research and development of new safe and effective TB vaccines.

One of the major limitations of current immunization strategies against TB is the vaccination route which may not be optimal for induction of protective immunity at the site of pathogen entry, that is, the respiratory tract. This has led to increased attention being directed toward mucosal immunization [9–14]. Mucosal delivery of BCG [9,10], the protein-based vaccines [11,12], and recombinant virus-vectored vaccines [13,14] enhance protection against TB. One explanation for the protection could be that mucosal vaccination induces trained innate immunity, tissue-resident memory T cells (T_RMs_), anti-TB surface antibodies, the effector cytokine IL-17 and inducible bronchus-associated lymphoid tissue (iBALT) in the lung [11,15].

However, the majority of antigens are not immunogenic and require strong adjuvants. Pulmonary vaccine delivery is limited by the fact that most mucosal adjuvants are unable to induce effective mucosal immunity or are too toxic [16]. Most of the current TB vaccine studies have focused on the effector cytokine IFN-γ or IL-17, and activation of Toll-like receptors (TLRs) to promote Th1 polarization [17]. Notably, activation of TLRs has been achieved via adjuvants such as CpG, MPLA, or Poly IC (polyinosinic-polycytidylic acid) [18]. CpG was vital for the protection generated by intramuscular CysVac2, but was dispensable for the protection induced by mucosal CysVac2 [11,19]. AS01, a MPLA-based adjuvant, has been tested in clinical trials [20]. However, mucosal MPLA failed to enhance protection against Mtb in mice [21]. Poly IC is a common subproduct during viral replication [22]. Mucosal Poly IC increases vaccine-induced CD8^+^ T cell immunity against influenza infection [23]. However, the efficiency of mucosal Poly IC in enhancing vaccine-induced protective immunity against TB is unknown.

Cationic antimicrobial peptides (AMPs) are essential defense components of the innate immune system, and play important protection roles against bacterial infection and immune regulation [24]. AMPs can trigger the adaptive immune response via activating antigen-presenting cells and influencing the production and polarization of lymphocyte responses [24]. LL-37, human AMP, administered orally could elicit antigen-specific IgA response [25]. Our previous study showed that pulmonary B5 (bovine neutrophil β-defensin-5, BNBD5) induced IgA response in airway [26]. However, the adjuvant activity of B5 in subunit vaccines and the mechanism of B5 regulating antigen-specific immune response have not yet been elucidated.

The main aim of this study was to determine the mucosal adjuvant potential of B5 and whether intranasal (i.n.) immunization with Poly IC and B5 (PB) promotes antigen-specific protective immunity against *M. bovis*. We designed a multistage fusion antigen, called AH, which contained the early-stage antigen Ag85A and the latency-associated antigen HspX, both are Mtb cell surface antigens. Ag85A-based viral vectored vaccines or adjuvant subunit vaccine have previously been used in human clinical trials [27]. Moreover, BCG vaccination failed to induce *T*-cell responses against HspX [28]. Thus, HspX was a promising TB vaccine target [29]. However, the efficacy of AH as a single antigen or as a booster vaccine component has not been investigated.

Here, this study shows that B5-based vaccine AH-PB via the intranasal route greatly enhanced CC-chemokine receptor 7 (CCR7) expression in the lung, leading to potent T cell and B cell priming and iBALT formation. Moreover, intranasal AH-PB greatly promoted T_RMs_ development and effector CD4 T cell proliferation in the lung, induced antigen-specific antibody responses in respiratory mucosa, provided similar protection against *M. bovis* compared to parenteral BCG, and boosted the protection induced by BCG. Furthermore, two AH-B5 fusion vaccines, AHB-P and pVAX1-AHB, also provide protection. These findings provide insights into B5 or other AMPs that can be optimized to amplify CD4 T cell and antibody response in the respiratory tract.

## Materials and methods

### Mice

All animal experiments and research protocols were approved by The Laboratory Animal Ethical Committee of China Agricultural University and the license number was AW91110202–2. The mice were purchased from Vital River Laboratories (Beijing, China) and were kept in the biosafety level 3 (BSL3) laboratory under specific pathogen-free conditions. During the study, mice received access to food and water ad libitum.

### Preparation of vaccines

B5 was prepared as previously described [26]. AH was generated through fusion of the genes of Ag85A and HspX with linker (G_4_S)_3_ using overlap polymerase chain reaction (PCR). PCR was used to amplify the DNA sequence of Ag85A and HspX from Mtb H37Rv chromosomal DNA because of the same amino acid sequences of the two genes from Mtb H37Rv and *M. bovis* AF2122/97, followed by splicing via overlap extension. The PCR product of Ag85A or AH was cloned into a pET30a (+) vector, while the PCR product of HspX was cloned into a pET28a (+) vector. Next, the plasmids pET30a (+)-Ag85A/AH and pET28a (+)-HspX were transferred into *E. coli* host BL21 (DE3). Two codon-optimized fusion gene Ag85A-(G_4_S)_3_-HspX-(G_4_S)_3_-B5 (AHB) sequences were synthesized at the Genewiz facility (Suzhou, China), the AHB sequence according to the codon preference of *E. coli* was subcloned into the pET30a (+) vector and transformed into *E. coli* host BL21 (DE3), another AHB sequence according to the codon preference of Bos taurus was subcloned into the pVAX1 vector and transformed into *E. coli* host DH5α. Ag85A, AH or AHB expressed as aggregated inclusion bodies, HspX expressed as a soluble component. The inclusion bodies were washed three times with wash buffer (pH 8.0) containing 20 mM Tris-HCl, 100 mM NaCl, 10 mM EDTA, and 0.5% tritonx-100 (V/V), and dissolved in binding buffer (pH 8.0) containing 8 M urea, 100 mM Na_2_PO_4_, and 10 mM Tris-HCl before being subjected to Ni-NTA affinity chromatography. After washing with binding buffer, the target proteins were washed with elution buffer (binding buffer supplemented with 250 mM imidazole). Next, the collected protein solution was gradually dialyzed against 20 mM Tris-HCl containing 1 mM EDTA, 10% glycerol, 1 mM GSH, 0.1 mM GSSG, and 0～8 M urea (pH 8.0), and finally dialyzed against PBS at 4℃. Notably, the soluble protein HspX was purified as previously described [26]. Finally, western blot using the mouse monoclonal His-tag antibody (Proteintech, Wuhan, Hubei, China) and Coomassie blue staining Solution (Solarbio, Beijing, China) were conducted to identify the proteins. The lipopolysaccharide in purified proteins was removed using ToxinEraser ^TM^ Endotoxin Removal Kit (GenScript, Nanjing, China). Residual lipopolysaccharide contamination was evaluated by the ToxinSensor ^TM^ Chromogenic LAL Endotoxin Assay Kit (GenScript, Nanjing, China) and determined to be < 20 endotoxin U/mg protein. The plasmid pVAX1- AHB was prepared using endotoxin-free plasmid kit (Omega Bio-tek Inc., Guangzhou, China). Human embryonic kidney 293T cells (10^5^ cells/well in 24-well plates) were transfected with 0.5 μg pVAX1- AHB or pVAX1 using lipofectamine™ 3000 transfection reagent (Thermo Fisher Scientific, Carlsbad, CA, USA). Following incubation for 24 h at 37°C with 5% CO_2_, whole-cell proteins were collected and tested by western blot with mouse polyclonal sera raised against AH (1:500).

### Immunization, challenge, and bacterial quantification

As shown in Figure S2. Female C57BL/6 mice, 6 to 8 weeks old, were immunized with subunit vaccines three times at three-week intervals. Intranasal (i.n.) immunizations comprised 50 μl of PBS containing 20 μg adjuvant [B5 and/or Poly IC (Sigma-Aldrich, St. Louis, MO, USA)] and/or 20 μg antigen, or containing 50 μg pVAX1 – AHB or pVAX1. Subcutaneous (s.c.) immunizations comprising 200 μl of AP containing 200 μg aluminum (alum) and 50 μg Poly IC alone or mixed with 40 μg AH were administered as effective adjuvant controls. Positive control mice received a single subcutaneous dose of 1 × 10^5^ colony-forming units (CFU) of BCG (Pasteur strain). In a prime-boost strategy, mice were subcutaneously immunized with 1 × 10^5^ CFU of BCG, rested for 4 weeks and then vaccinated with AH-PB or AH-AP at three-week intervals. Mice were challenged with *M. bovis* (Beijing strain C68004) 3 weeks after the last immunization via the i.n. route at 100–500 CFU per mouse. Three mice were enthanized 24 h after challenge to determine the initial bacterial load in the lung. Next, mice were enthanized 4 or 8 weeks after challenge, and bacterial quantification was performed by plating serial dilutions of lung and spleen homogenates on 7H10 (Difco, New York, NY, USA) agar plates supplemented with 10% oleic acid-albumin enrichment (BD Biosciences, New York, NY, USA), amphotericin B (50 µg/mL) and polymyxin B sulfate (50 µg/mL), then the agar plates were cultured for 3–4 weeks at 37°C.

### Antibody and cytokine ELISAs

Blood and bronchoalveolar lavage fluid (BALF) samples were collected 3 or 6 weeks after the final immunization using a previously described protocol [30]. Next, the level of IgG, IgA, or IgM response to antigen was assessed. Briefly, ELISA plates were coated with 0.5 µg antigen (AH, Ag85A, HspX, or PPD) overnight at 4°C followed by blocking with 5% fat-free dry milk for 2 h at 37°C and washing three times with PBS/Tween 20 (0.05%). 100 µl of serially diluted serum (1:10, six dilutions per sample) or BALF (1:500 dilution for IgG measurement) was incubated for 1.5 h at 37°C followed by washing. Next, 100 µl of goat anti-mouse HRP-conjugated IgG, IgA, or IgM (1:2000 dilution) (Qiyi Biological Technology Co., Ltd., Shanghai, China) was added for 1.5 h at 37°C followed by washing. 100 µl of TMB (3, 3′,5,5′-tetramethylbenzidine) substrate was then added for 15 min, followed by 100 µl of sulfuric acid (2 N). Data were collected on ELISA Plate Reader at 450 nm and the level of IgG in serum were presented as endpoint titer with a cutoff of 0.2 [31]. The total IgA concentration in BALF was determined using an ELISA kit (Neobioscience, Guangdong, China) according to the manufacturer’s instructions. Splenocytes were isolated 3 weeks after the last immunization. The prepared splenocytes were stimulated with AH or PPD (10 μg/mL) in 24-well U bottom plates for 24 h at 37°C. Finally, the cell culture supernatants were collected to measure the concentrations of IFN-γ and IL-17 using ELISA kits (Neobioscience) according to the manufacturer’s instructions.

### Flow cytometry

Staining for intracellular cytokine: cells isolated from lung tissues were stimulated with AH (10 μg/mL) for 812 h at 37°C followed by addition of the brefeldin A/monensin mixture. Subsequently, the cells were incubated with anti-CD16/32 for 10 min at 4℃ to block Fc receptors, washed and stained with (PerCP)-Cy5.5-CD3ε and PE-cy7-CD4 for 30 min at 4℃. Cells were fixed and permeabilized with FIX & PERM Kit. Next, cells were stained with PE-IL-17 and FITC-IFN-γ for 30 min at 4°C Staining for cell surface: cells isolated from lung tissues or BALF were incubated with fluorochrome-labeled antibodies against CD3ε, CD4, CD8α, CD44, CD69, and CD103 (Figure S7) for 30 min at 4°C. The stained cells were washed and then fixed with 4% paraformaldehyde. Finally, cells were analyzed using a FACSVerse flow cytometer (BD Biosciences) and a commercially available FlowJo software (Figure S7). Antibodies and reagents for flow cytometry were from MultiSciences (Hangzhou, China).

### Histology and immunohistochemistry

Heart, liver, kidney, spleen, and lung tissues were fixed in 10% normal buffered formalin, and then embedded in paraffin, cut into 3-μm sections, deparaffinized, and stained with hematoxylin and eosin (HE) stain or Kinyoun acid-fast stain (Leagene, Beijing, China). The HE-stained lung tissue sections were scanned using a VENTANA DP 200 slide scanner (Roche Diagnostics, Rotkreuz, Switzerland). Qualitative analyses were performed using ImageJ software for evaluation of inflammatory area in lungs. For immunohistochemistry (IHC) analysis, the 3-μm sections were deparaffinized, performed heat-induced antigen retrieval with sodium citrate solution containing 10 mM trisodium citrate dihydrate and 1.9 mM citric acid, followed by washing three times with PBS and blocking with 3% H_2_O_2_ for 30 min at room temperature, the sections were washed and blocked with normal goat serum for 1 h at room temperature. Then the sections were incubated with rabbit anti-CD3 (1:1000, from Proteintech), anti-CD19 (1:200, from Solarbio) and CCR7 (1:200, from Proteintech) for 12–18 h at 4°C and then washed and incubated with horseradish peroxidase (HRP)-conjugated goat anti- rabbit secondary antibody for 30 min at 37°C DAB (3,3N-diaminobenzidine tertrahydrochloride) was then added for 3–5 min, followed by staining with hematoxylin.

### Statistical analysis

All data are expressed as mean ± standard deviation (SD). For comparison between two groups, Student’s *t*-test was applied and for comparisons among more than two groups, one-way ANOVA followed by the Tukey’s multiple-comparison test using GraphPad Prism version 7.0 software was performed. Flow cytometry results were analyzed using the FlowJo software version 10. A p-value less than 0.05 was considered statistically significant.

## Results

### Intranasal AH-PB induces iBALT formation in the lung and T cell proliferation in airway

**Figure 1.**
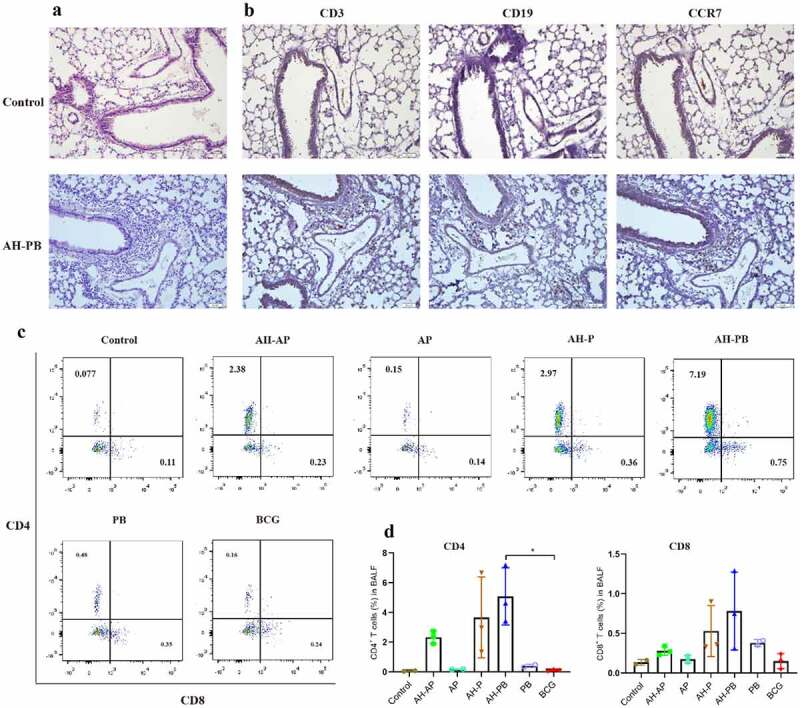
Intranasal AH-PB induces the generation of T cells and B cells in the lung. Mice (n = 3) were vaccinated through i.n. route with AH-P, AH-PB or PB, or s.c. route with AH-AP, AP or BCG (three times, 3 weeks interval). Lungs and bronchoalveolar lavage fluid (BALF) were collected three weeks after the last immunization. (a) Histopathological examination of lung tissues via hematoxylin and eosin (HE) staining (scale bar: 50 μm). (b) Immunohistochemical (IHC) examination of lung tissues (scale bar: 50 μm). The photos were from the same area of the same lung tissue section with different antibody staining. (c) Representative FACS blots of CD4^+^ and CD8^+^ T cells in BALF. (d), Percentage of CD4^+^ and CD8^+^ T cells in BALF. Data shown are means ± SD. The significance of differences between the groups was determined by ANOVA with post-hoc Tukey’s multiple comparison test (*p < 0.033).

We purified the recombinant fusion proteins AH (～54 kDa, >95% purity) and B5 (～7 kDa, >95% purity) (Figure S1), and then evaluated safety and immunogenicity of each vaccination strategy (Figure S2A). Alum mixed with Poly IC (AP) could promote a TB subunit vaccine-generated protection [32], thus AP was used as an effective adjuvant control. Our results showed that almost all vaccinated-mice exhibited obvious weight gain. The heart, kidney, and spleen isolated from all vaccinated-mice were normal. However, a small amount of inflammation foci was generated in livers from mice administrated with AP or BCG. Moreover, intranasal AH-PB induced an increased number of lymphoid cells accumulating around the bronchi and blood vessels in the lung, but the number of inflammatory cells in lungs gradually decreased, and only a small number of inflammatory cells appeared 6 and 9 weeks after vaccination (**Figure 1(a)** and Figure S3E), indicating that the inflammatory changes were mild and temporary. To further investigate the intranasal AH-PB-induced inflammatory cells in the lung, these cells were analyzed through IHC staining. IHC results showed that the AH-PB-induced inflammatory cells in the lung were mainly CD3^+^ T cells and CD19^+^ B cells, and highly expressed CCR7 (Figure 1(b)), which was in line with the results showing that mucosal vaccination induced formation of iBALT characterized by accumulation of T and B cells in previous studies [11,33]. Moreover. AH-PB and AH-P increased the number of CD4^+^ and CD8^+^ T cells in BALF (Figure 1(c,d)). Collectively, these results suggest that intranasal AH-PB induces the formation of iBALT in the lung and the proliferation of T cells in airway.

### Intranasal AH-PB induces antigen-specific antibody response in airway

**Figure 2.**
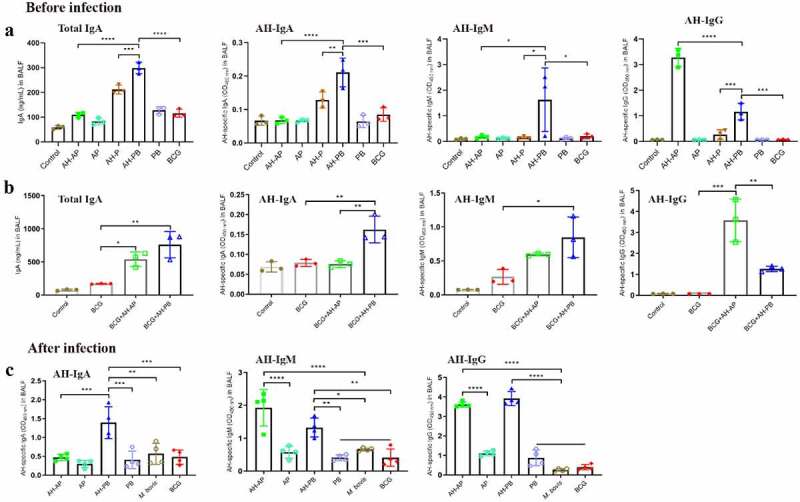
Intranasal AH-PB induces antigen-specific antibody response in BALF. Mice or BCG-immunized mice were vaccinated through i.n. route with AH-P, AH-PB or PB, or s.c. route with AH-AP, AP or BCG (three times, 3 weeks interval). Three weeks after the last immunization, mice were infected with *M. bovis*. BALF were collected three weeks after the last immunization and eight weeks after challenge. (a) and (b) Levels of total IgA, AH-specific IgA, IgM and IgG in BALF before infection. (c) Levels of AH-specific IgA, IgM and IgG in BALF after infection. The BALF samples for IgG measurement were diluted 500 times with PBS. Data shown are means ± SD. Data are representative of three independent experiments (n = 3–4 mice per group). The significance of differences between the groups was determined by ANOVA with post-hoc Tukey’s multiple comparison test (*p < 0.033, **p < 0.0021, ***p < 0.0002, ****p < 0.0001).

The antibody-mediated immune response in BALF was evaluated 3 weeks after the last immunization. Results showed that levels of total IgA, AH-specific IgA, IgM, and IgG in BALF of the AH-PB group were significantly higher than that of the AH-P and AH-B group (Figure 2(a) and Figure S4B). Moreover, AH-PB could also induce similar antibody response in BCG-immunized mice (Figure 2(b)), and still induce high level of AH-specific antibody response after infection (Figure 2(c)). However, AH-AP only induced high level of AH-specific IgG response in airway. After challenge, AH-AP promoted the AH-specific IgM but not IgA response, while AH-PB promoted both IgA and IgG but not IgM response (Figure 2). In addition, both AH-AP and AH-PB induced high level of AH-specific IgG response in serum, and the serum against AH could recognize Ag85A, HspX and PPD (Figure S4A). Overall, these findings indicate that intranasal AH-PB induces a strong antigen-specific antibody response in respiratory mucosa and B5 enhances the immune response.

### Intranasal B5 is crucial for the efficacy generated by AH-PB

**Figure 3.**
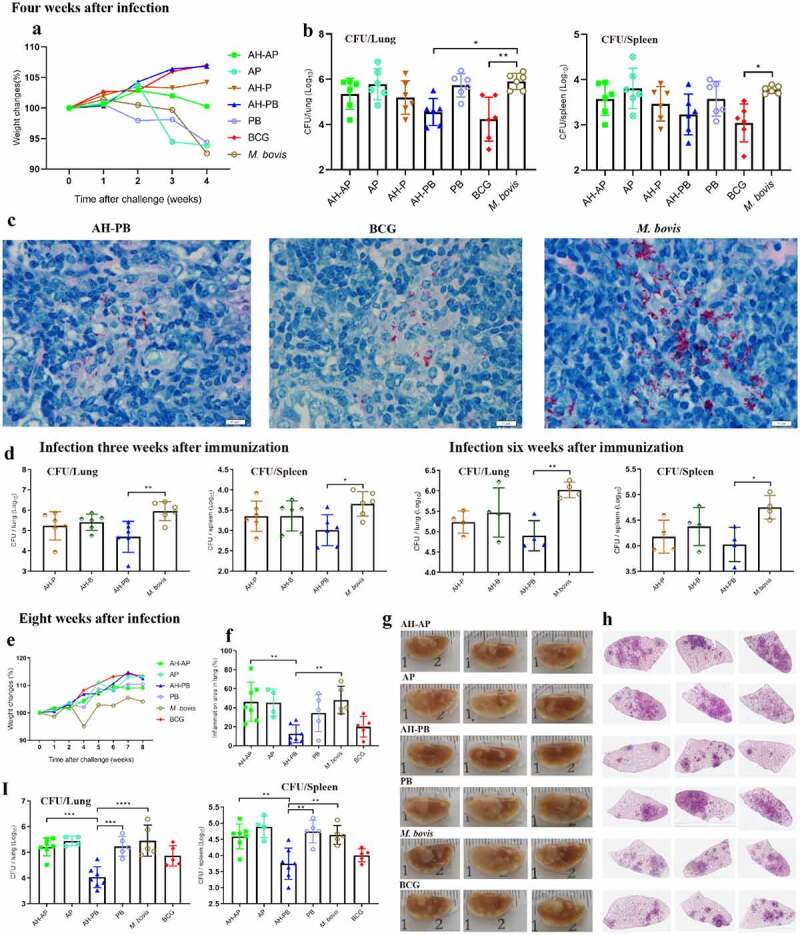
Intranasal AH-PB provides protection against *M. bovis*. Mice were vaccinated through i.n. route with AH-P, AH-B, AH-PB or PB, or s.c. route with AH-AP, AP or BCG (three times, 3 weeks interval). Three or six weeks after the last immunization, mice were infected with *M. bovis*. Mice were euthanized and protective efficacy was assayed four or eight weeks after challenge. (a) Weekly weight monitoring after *M. bovis* challenge. (b) Bacterial burden in lungs and spleen. (c) Lung tissues were performed with acid-fast staining (scale bar: 10 μm). (d) Bacterial load in lungs and spleen. (e) Weekly weight monitoring after *M. bovis* challenge. (f) Percentage of pulmonary inflammation area. (g) Gross pathology of formalin-fixed left lungs. (h) Scanning images of left lung lobe with HE staining. (i) Bacterial burden in lungs and spleen. Data shown are means ± SD. Data are representative of two independent experiments (n = 4–7 mice per group). The significance of differences between the groups was determined by ANOVA with post-hoc Tukey’s multiple comparison test (*p < 0.033, **p < 0.0021, ***p < 0.0002, ****p < 0.0001).

The efficacy of AH-PB vaccine was subsequently tested (Figure S2A). Four weeks after challenge, the weight of body, lungs, and spleen revealed that AH-PB alleviated the degree of pathogenesis of *M. bovis*-infectedmice compared to PBS/PB-treated mice (Figure 3(a) and Figure S5A). More importantly, the lung bacterial load was significantly reduced by AH-PB but not AH-P or AH-AP, consistent with this, a significant decrease in red acid-fast bacilli (AFB) was observed in the AH-PB group. However, AH-PB had no significant effect on the spleen bacterial load at week 4 after challenge (Figure 3(b,c)). In another independent experiment (Figure S2B), 8 weeks after challenge, AH-PB maintained the ability of alleviating the degree of pathogenesis caused by *M. bovis* (Figure 3(e,g)) and Figure S5B). Moreover, the *M. bovis* group and the PB group showed appearance of widespread visible gray-white nodular lesions and large area of inflammatory foci in left lungs. However, fewer lung nodular lesions and inflammatory foci were observed in AH-PB group and BCG group (Figure 3(f–h)). Furthermore, both lung and spleen bacterial load in the AH-PB group were significantly reduced compared to the *M. bovis* group (Figure 3(I)). It was worth noting that the bacterial burden and the inflammatory foci in AH-PB group was less than that in the BCG control group, suggesting that the protection of AH-PB is better than that of BCG in the late stage of infection. Moreover, AH-PB, but not AH-P or AH-B, significantly decreased the bacterial burden in lungs and spleen compared to the *M. bovis* control (Figure 3D). In addition, four independent experiments in this study showed that AH-PB resulted in a decrease in lung CFU of 1.4 log10 and spleen CFU of 0.9 log10 compared to the *M. bovis* control (Figure S5F). Overall, these results suggest that both B5 and Poly IC are crucial for the protection generated by AH-PB and this protection correlates with IFN-γ-producing CD4^+^ T cells.

### Intranasal AH-PB boosts the protective efficacy induced by BCG

**Figure 4.**
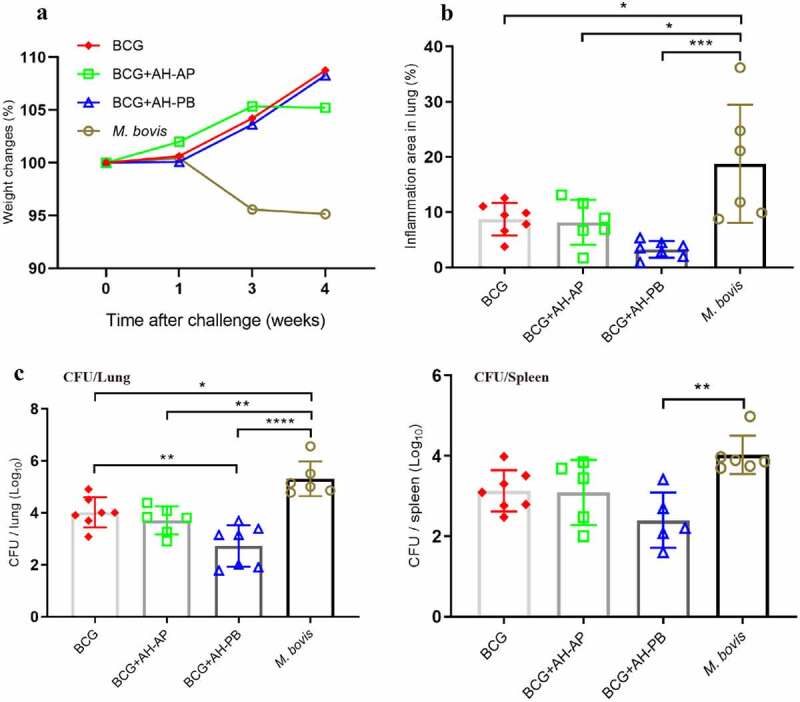
Intranasal AH-PB boosts the protective efficacy induced by BCG. BCG-immunized mice were vaccinated through i.n. route with AH-PB or s.c. route with AH-AP (three times, 3 weeks interval). Three weeks after the last immunization, mice were infected with *M. bovis*. Four weeks after challenge, mice were enthanized to evaluate the protective efficacy. (a) Weekly weight monitoring after *M. bovis* challenge. (b) Percentage of pulmonary inflammation area. (c) Bacterial burden in lungs and spleen. Data shown are means ± SD. Data are representative of two independent experiments (n = 6–7 mice per group). The significance of differences between the groups was determined by ANOVA with post-hoc Tukey’s multiple comparison test (*p < 0.033, **p < 0.0021, ***p < 0.0002, ****p < 0.0001).

To demonstrate the efficacy of AH-PB in a prime-boost strategy, BCG-immunized mice were vaccinated with AH-PB (Figure S2D). It was found that BCG-immunized mice gained weight, while PBS-immunized mice lost weight after *M. bovis* infection (Figure 4(a)). AH-PB booster immunization could not ameliorate enlarged lung and spleen tissues compared to BCG (Figure S5C); however, it significantly reduced the percentage of pulmonary inflammation area (Figure 4(b)) and the bacterial burden in lungs and spleen (Figure 4(c)). In addition, one out of six mice in the BCG + AH-AP group and two out of seven mice in the BCG + AH-PB group exhibited no detectable bacteria in spleen. Moreover, AH-PB provided better efficacy than AH-P in the prime-boost strategy (Figure S5E), suggesting that B5 promoted vaccine-induced protection. However, AH-AP booster immunization failed to enhance the protection induced by BCG. In addition, we found that AH-PB immunization twice could also boost the protection generated by BCG (Figure S5 H). Overall, these results indicate that intranasal AH-PB is able to boost the protective efficacy of BCG.

### Intranasal AH-PB induces the generation of tissue-resident and effector CD4 T cell in the lung

**Figure 5.**
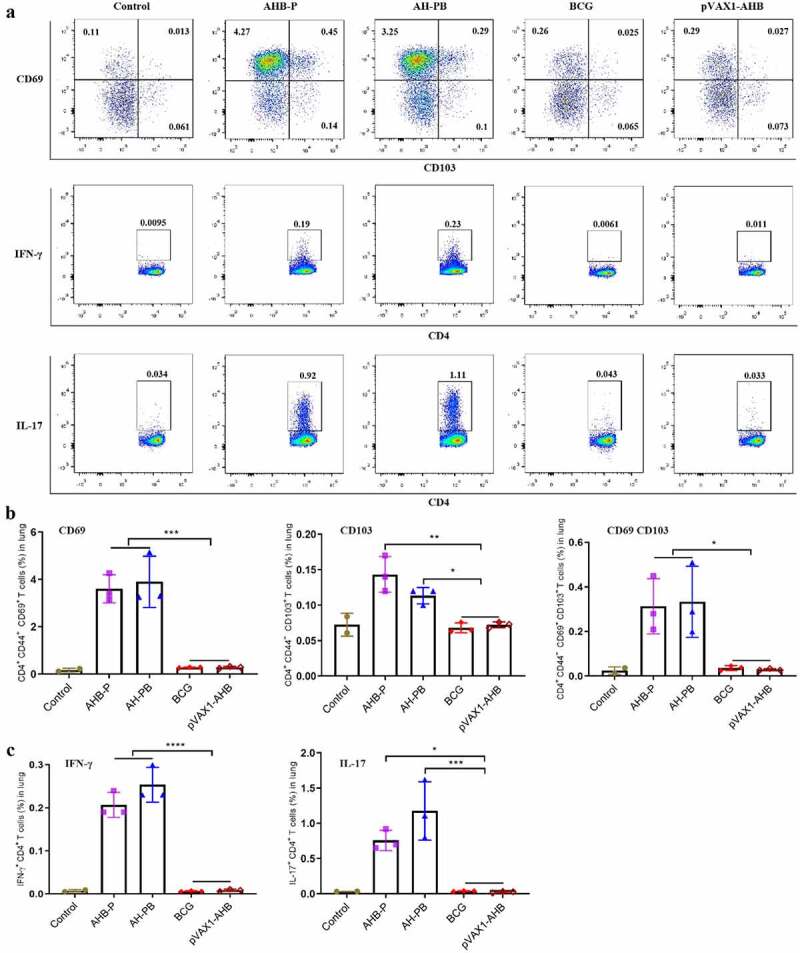
Intranasal AH-PB promotes the generation of the T_RMs_ and multi-cytokine-producing CD4^+^ T cells. Mice were vaccinated through i.n. route with AHB-P, AH-PB or pVAX1-AHB, or s.c. route with BCG (three times, 3 weeks interval). Lung tissues were collected three weeks after the last immunization. (a) Representative FACS blots of T_RMs,_ IFN-γ-producing and IL-17-producing CD4^+^ T cells in lungs. (b) Percentage of CD44^+^ CD4^+^ T cells expressed CD69, CD103 or both. (c) Percentage of IFN-γ or IL-17-producing CD4^+^ T. Data are representative of two independent experiments (n = 3 mice per group). The significance of differences between the groups was determined by ANOVA with post-hoc Tukey’s multiple comparison test (*p < 0.033, **p < 0.0021, ***p < 0.0002, ****p < 0.0001).

We next evaluated the ability of AH-PB to prime lung parenchyma-homing T cells. CD44 is a marker of activated and memory T cells [34]. In this study, the majority of lung CD44^+^ CD4^+^ T cells induced by intranasal AH-PB highly expressed the T_RM_ markers CD69, CD103, or both, whereas T cells generated by BCG expressed these markers at a much lower frequency (Figure 5(a,b)). Meanwhile, AH-PB markedly increased the number of CD44^+^ CD4^+^ T cells in BALF (Figure S6A). Moreover, AH-PB significantly increased the number of IFN-γ or IL-17-producing CD4^+^ T cells in lungs (Figure 5(a,c)) and Figure S4C). In addition, AH-PB significantly promoted secretion of AH/PPD-specific IFN-γ and AH-specific IL-17 in splenocytes (Figure S6B). Collectively, these results suggest that intranasal AH-PB promotes T_RM_ development and induces multi-cytokine-secreting CD4^+^ T cells generation in the lung.

### AH-B5 fusion vaccines provide protection

**Figure 6.**
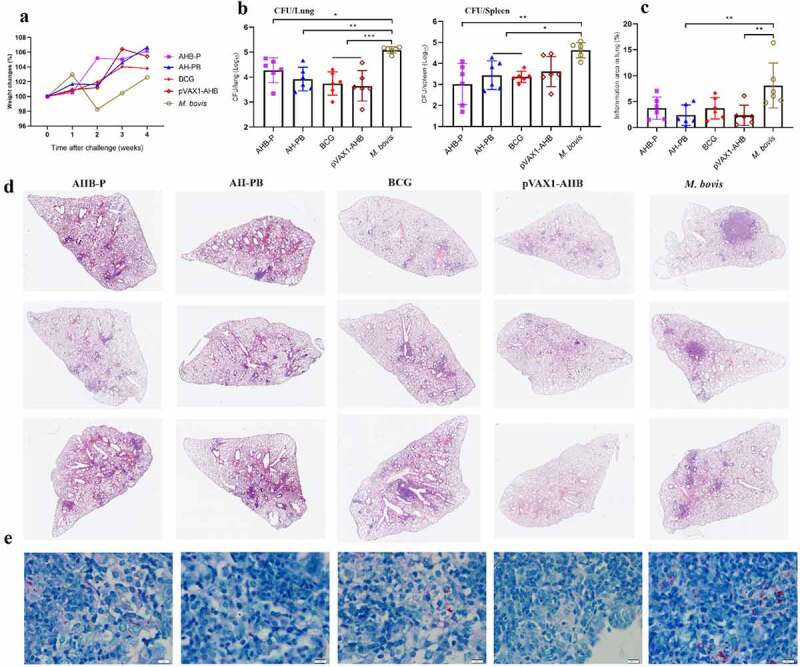
AH-B5 fusion vaccines provide protection. Mice were vaccinated through i.n. route with AHB-P, AH-PB or pVAX1-AHB, or s.c. route with BCG (three times, 3 weeks interval). Three weeks after the last immunization, mice were infected with *M. bovis*. Mice were euthanized to assess protective efficacy four weeks after challenge. (a) Weekly weight monitoring after *M. bovis* challenge. (b) Bacterial burden in lungs and spleen. (c) Percentage of pulmonary inflammation area. (d) Scanning images of left lung lobe performed with HE staining. (e) Lung tissues was performed with acid-fast staining (scale bar: 10 μm). Data shown are means ± SD. Data are representative of two independent experiments (n = 6 mice per group). The significance of differences between the groups was determined by ANOVA with post-hoc Tukey’s multiple comparison test (*p < 0.033, **p < 0.0021, ***p < 0.0002).

In order to simplify the preparation process of AH-PB, we co-expressed AH and B5, and prepared AH-B5 fusion vaccines AHB-P and pVAX1-AHB (Figure S1), and assessed their immunogenicity and protective efficacy (Figure S2E). Results showed that intranasal AHB-P but not pVAX1-AHB promoted the production of T_RMs_, IFN-γ or IL-17-producing CD4^+^ T cells in lungs and CD44^+^ CD4^+^ T cells in BALF, and facilitated the secretion of AH-specific IFN-γ and IL-17 in splenocytes, suggesting that AHB-P could also induce similar T cell immune response in lungs and spleen in comparison with AH-PB. However, AHB-P induced a low level of antigen-specific antibody response (Figure 5 and Figure S6). Interestingly, both AHB-P and pVAX1-AHB provided protection by reducing the bacterial load and alleviating the pulmonary pathological lesions of the *M. bovis*-infected mice, and exhibited similar protective efficacy in comparison with AH-PB (Figure 6, Figure S5D). Moreover, intranasal pVAX1-AHB significantly diminished the bacterial load in lungs and spleen compared to pVAX1 (Figure S5 G). Overall, these results suggest that intranasal AH-B5 fusion vaccines also provide protection against *M. bovis*.

## Discussion

The efficacy and safety of respiratory mucosal adjuvants limits further research on mucosal vaccines. For example, mucosal CpG or MPLA mixed with Ag85A induced Th1 and Th17 responses, but only conferred marginal protection against Mtb [21]. Intranasal LTK63-adjuvanted H1 generated high levels of protection [12], however, the appearance of two cases of transient facial paralysis during clinical trials restricted further use of LTK63 [35]. Other adjuvants such as CpG [11,21], MPLA [21], and CAF01 [36] used for parenteral immunization provided insignificant protection in mucosal strategies. Therefore, this calls for the development of safe and effective mucosal adjuvants. Herein, PB containing Poly IC and B5 was a safe and effective mucosal adjuvant, promoted respiratory antigen-specific antibody response and memory *T*-cell response, enhanced vaccine-induced protection against *M. bovis*, and provided better protection than BCG in the later stage of *M. bovis* infection.

Mucosal protein/adjuvant or BCG induced inflammatory changes including the formation of iBALT in the lung [10,11,37]. The iBALT formation was thought to be associated with protection [11,33]. In this study, intranasal AH-PB induced iBALT in the lung, but this inflammatory response was temporary and mild compared to intratracheal BCG [37]. This suggests that mucosal AH-PB exhibited the potential advantage of security.

Antibody-mediated immunity in TB has been neglected, but there is emerging evidence supporting a role for antibodies in protection against Mtb, thus a growing interest focuses on determining their relevance to vaccine development [38,39]. Mtb cell surface-neutralizing antibodies were shown to be associated with enhanced protection [40]. Moreover, antigen-specific IgA, IgM and IgG responses were thought to be related to protection [39,41]. And intranasal B5 could induce IgA response in airway [26]. Therefore, based on these findings, we chose two Mtb surface antigens to prepare AH-PB with the overarching goal of inducing antigen-specific antibody response in airway. As expected, intranasal AH-PB induced high levels of antigen-specific IgA, IgM, IgG, and B5 amplified this antibody response. More importantly, AH-PB provided promising protection from 100–500 CFU of *M. bovis* challenge, and also boosted protection induced by BCG. Like other tuberculosis vaccines [9–14,33,36], AH-PB vaccination failed to eradicate bacteria at week 4 and 8 after challenge in mice. This may be associated with infection dose. In the standard mouse TB model, mice are infected with 50–100 CFU. However, the typical human infection dose is much lower (1–3 bacilli) in most cases [42]. Lower doses (<15 CFU) have been allowed to investigated TB vaccine-mediated sterilizing immunity [31,41]. Moreover, given the fact that the sequence similarity of Mtb and *M. bovis* is more than 99.9% and the amino acid sequences of Ag85A and HspX from Mtb H37Rv and *M. bovis* AF2122/97 are the same [2], intranasal AH-PB may provide protection against Mtb. Although AH-AP induced a high level of AH-specific IgG response in airway, it did not provide protection in the lung, suggesting that IgG response may not play a critical role in host defense, or mucosal vaccine-induced protection may be related with cooperation of multiple immune molecules in respiratory tract.

To simplify the preparation process of AH-PB, AHB-P and pVAX1-AHB were prepared by co-expressing AH and B5. Interestingly, they both conferred protection against *M. bovis*, and their protective efficacy was equivalent to AH-PB and BCG. However, it was difficult to explain the mechanism of protection induced by pVAX1-AHB, unlike AHB-P, pVAX1-AHB failed to induce T cell immunity in lungs and antibody response in airway, but still provided protection. It is likely that other immunity mechanisms mediated the protection. Overall, we demonstrated that B5 is a potential effective mucosal adjuvant used in TB vaccines, and intranasal Poly IC could promote protection against *M. bovis*.

Antibodies that mediated protection were dependent on CD4^+^ T cells for efficacy [40], and T cells are essential for controlling TB. Although IFN-γ-producing CD4^+^ T cells were reported to be irrelevant to protection [11,36,43], Th1 cell response is still used as a key criterion for TB vaccine. In this study, intranasal AH-PB strongly induced IFN-γ-producing CD4^+^ T cells generation in the lung. Interestingly, AH-PB elicited protection, while mucosal AERAS-402 [43] or H56:CAF01 [36] failed to provide protection. This suggests that IFN-γ-producing CD4^+^ T cell response is not the sole effector mechanism. IL-17 is important for controlling Mtb [11,44]. IL-17-producing CD4+ T cells in the lung were strongly increased after AH-PB intranasal delivery. Moreover, CD4 T_RMs_ play an important role in protection from many pathogens, including Mtb [45–47]. Therefore, strategies to promote T_RM_ generation are of great interest for TB control. As expected, mucosal AH-PB or AHB-P greatly promoted T_RM_ development in the lung. Overall, our findings support the notion that at least two mechanisms mediated protection induced by AH-PB or AHB-P: cell-mediated immunity and humoral immunity in respiratory tract.

The novel immunization strategy in this study induced strong protective immune responses, and the significance of our findings was dependent on the route of immunization. BCG is effective, but it causes severe inflammatory response in the lung [37,48]. However, AH-PB, a less cytotoxic mucosal vaccine, provides a strategy because it could be directly administered into the respiratory tract and conferred significant protection against *M. bovis*. Moreover, antigen-defensin fusion vaccines were feasible, cost-effective and protective. It was conducive to the control of tuberculosis in economic animals like cattle. Some TB vaccine candidates such as MVA85A [49] that could protect mice but failed to protect human or cattle. MVA85A containing a single antigen Ag85A only induced cellular immune response, while AH-PB containing Ag85A and latency-associated antigen HspX as well as adjuvants (Poly IC and B5) induced all-around respiratory mucosal immune response including T cell response, antibody response and trained immunity (data not shown). Nonetheless, further studies will be needed to evaluate the safety and protection in cattle. Moreover, AMPs from animals and plants were suggested to therapeutically apply to humans, thereby restricting the risk of evolved cross-resistance to endogenous host defense [50]. Thus, it is possible to use B5 in human. Overall, this study has offered a possibility that B5 or other AMPs can be used as mucosal adjuvants and in the development of mucosal vaccines against TB or other respiratory diseases.

## Data Availability

The data that support the findings of this study are available from the corresponding author upon reasonable request (zhouxm@cau.edu.cn).
